# The WD40 Domain Is Required for LRRK2 Neurotoxicity

**DOI:** 10.1371/journal.pone.0008463

**Published:** 2009-12-24

**Authors:** Nathan D. Jorgensen, Yong Peng, Cherry C.-Y. Ho, Hardy J. Rideout, Donald Petrey, Peng Liu, William T. Dauer

**Affiliations:** 1 Department of Neurology, Columbia University Medical Center, New York, New York, United States of America; 2 Department of Biochemistry and Molecular Biophysics, Columbia University Medical Center, New York, New York, United States of America; 3 Department of Neurology, University of Michigan Medical School, Ann Arbor, Michigan, United States of America; 4 Department of Cell and Developmental Biology, University of Michigan Medical School, Ann Arbor, Michigan, United States of America; National Institutes of Health, United States of America

## Abstract

**Background:**

Mutations in leucine-rich repeat kinase 2 (LRRK2) are the most common genetic cause of Parkinson disease (PD). LRRK2 contains an “enzymatic core” composed of GTPase and kinase domains that is flanked by leucine-rich repeat (LRR) and WD40 protein-protein interaction domains. While kinase activity and GTP-binding have both been implicated in LRRK2 neurotoxicity, the potential role of other LRRK2 domains has not been as extensively explored.

**Principal Findings:**

We demonstrate that LRRK2 normally exists in a dimeric complex, and that removing the WD40 domain prevents complex formation and autophosphorylation. Moreover, loss of the WD40 domain completely blocks the neurotoxicity of multiple LRRK2 PD mutations.

**Conclusion:**

These findings suggest that LRRK2 dimerization and autophosphorylation may be required for the neurotoxicity of LRRK2 PD mutations and highlight a potential role for the WD40 domain in the mechanism of LRRK2-mediated cell death.

## Introduction

PD is a neurodegenerative disease characterized by tremor, rigidity, akinesia, and postural instability [1] that affects 4% of the population over the age of 65 [2]. The economic impact ranges from 13 to 29 billion dollars annually in the US alone [3]. All current treatments for PD act by suppressing disease symptoms; none slow or prevent the underlying neurodegenerative process. Incomplete understanding of the molecular mechanisms that mediate neurodegeneration in PD has limited the development of neuroprotective drugs.

Mutations in a growing list of genes have been linked to the pathogenesis of PD [4], [5], providing valuable clues into the pathogenic mechanisms of the disease [6]. LRRK2 is one of these, and multiple aspects of LRRK2 biology have combined to create considerable interest in this protein. First, LRRK2 mutations are the most common genetic cause of PD [4]. LRRK2 mutations account for approximately 5% of familial and 2% of sporadic PD [7], [8]. Second, most patients with LRRK2 mutations exhibit clinical and pathological features that are indistinguishable from idiopathic PD [9]. Finally, the well-defined catalytic domains present in LRRK2 render functional assays on this molecule tractable, and suggest that it may be amenable to therapeutic targeting.

**Figure 1:**
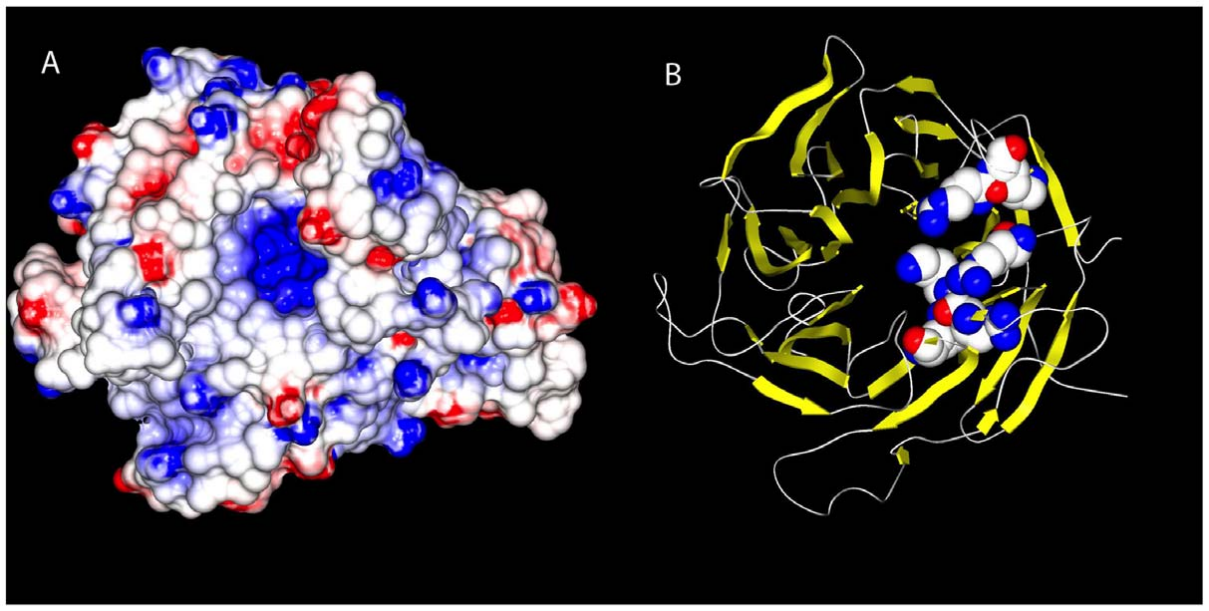
LRRK2 C-terminus forms distinct beta-propeller configuration. Molecular surface of the homology model of the WD40 domain of LRRK2 (left) using the structure of the BUB3 mitotic checkpoint protein (PDB code 1yfq) as a template. The coloring of the surface is determined by the electrostatic potential at each point on the surface (red = acidic, white = neutral and blue = basic). The prominent basic cleft shown at the center of the molecule was consistently present in other models of the WD40 domain of LRRK2 based on other templates. The basic character of this cleft was due to a set of basic residues that were consistently placed in each of the models we examined (K2367, R2413, K2415, R2456, R2477 and K2478). A ribbon diagram of the same model is shown at the right, highlighting these residues in sphere representation. These residues were consistently clustered together in all of the models examined. Similar modeling of LRRK1 C-terminus failed, highlighting previously suggested divergence between LRRK1 and LRRK2 in this region.

LRRK2 is a complex 286 kDa protein that contains multiple well-recognized domains, including (in order, from amino to caboxyl terminus): LRR, Ras of complex (ROC), carboxyl-terminus of ROC (COR), kinase and WD40 domains (Figure 1A). Multiple studies have focused on the functions of the ROC and kinase domains [10], [11], [12], [13]. LRRK2 isolated from murine brain possesses GTPase activity, but this activity is considerably lower when LRRK2 is isolated from other tissues [13], [14]. GTP binding stimulates LRRK2 kinase activity, potentially linking the ROC domain to the activity of the kinase domain [14]. *In vitro* assays demonstrate that LRRK2 can both autophosphorylate (via an intramolecular process) as well as trans-phosphorylate proteins such as moesin, 4E-BP, and myelin basic protein (MBP) [15], [16], [17], [18]. Evidence from the *Dictyostelium* LRRK2-homolog suggests that the COR domain acts as a hinge to transduce an intramolecular signal between the ROC and the kinase domain providing a potential mechanism for ROC's regulation of LRRK2 kinase activity [19]. Cookson and colleagues have also suggested that the COR functions as a hinge based on their analysis of the human LRRK2 COR and ROC domains [20].

Mutations in the kinase, COR, and ROC domains segregate with PD in large family studies [21]. Biochemical analyses of these mutations have focused on the kinase activity of LRRK2. One mutation in the kinase domain (G2019S; figure 1A) increases kinase activity approximately three-fold while other pathogenic mutations have an effect on kinase activity ranging from 0–50% [11], [16], [17]. Notably, disrupting the kinase activity by mutating a conserved lysine in the kinase domain that mediates ATP binding eliminates the neurotoxicity of LRRK2 [10], [11]. Interestingly, placing LRRK2 PD mutations in the corresponding residues of LRRK1, LRRK2's closest homologue, fails to enhance the ability of this molecule to effect cell death [22].

Some evidence indicates that LRRK2 may exist as a dimer. LRRK2 interacts with itself, as shown by immunoprecipitation of differentially tagged LRRK2 molecules [18], [23]. Crystallization of LRRK2 fragments containing the ROC domain found that it forms a ROC-ROC dimer [20], and studies in a related protein from the bacteria *C. tepidum* suggest that the adjacent COR domain dimerizes [24]. Subsequently, a significant fraction of LRRK2 was found to migrate in a ∼600 kDa complex on blue native gels and on a size-exclusion gel-filtration column, with the remainder migrating as a much larger complex [18]. These studies did not further analyze the composition of these complexes, leaving open the possibility that they contained LRRK2 complexed with other proteins (e.g. HSP90, or FADD), rather than homo-multimers of LRRK2. It is also unknown whether the presence of LRRK2 in these complexes is related to its ability to effect neuronal death.

The contribution of the LRR and WD40 protein-protein interaction domains to LRRK2 function has not been extensively explored, yet either or both of these may contribute to the proper formation of LRRK2-containing complexes. Notably, a sequence variation (G2385R) in the WD40 domain has been implicated as a risk factor for PD [25], [26], [27]. In addition, the WD40 domain is of particular interest, as this is a region of particular divergence between LRRK1 and LRRK2. Multiple observations [21], [28] indicate the LRRK1 may not contain a *bone fide* WD40 domain, potentially contributing the failure of LRRK2 PD mutations to enhance the neurotoxicity of LRRK1 [22]. Based on these observations, we undertook a detailed study of the WD40 domain of LRRK2, exploring its potential involvement in LRRK2 complex formation, kinase function and ability to cause neuronal cell death.

## Results

### Homology Modeling of the WD40 Domain

The failure of LRRK2 PD mutations to alter the neurotoxicity of LRRK1 and the apparent divergence between C-terminal regions of LRRK2 and LRRK1 led us to model and compare this region of the two proteins. We began by using homology modeling to assess the structure of residues 2101-2527 of LRRK2. Modeling templates with E-values ranging from 10^20^ to 10^24^ were identified in the Protein Data Bank [42] using the profile-profile alignment program HMAP [41]. Models based on each template were generated using NEST software [29]. The top four models were TUP1 (PDB code 1erj), actin interacting protein 1 (1nr0), cytosolic Fe-S assembly protein 1 (2hes), BUB3 mitotic checkpoint protein (1yfq). All four models produced a similar structure of a beta-propeller cylinder repeat with a cleft composed of basic residues typical of WD40 domains (Figure 1).

A similar search of the non-redundant sequence database (nr) using residues 1561–2038 of LRRK1 did not identify any similarity of the LRRK1 carboxyl-terminus to LRRK2 or any of its close homologs when 5 iterations of PSI-BLAST with an E-value cutoff of 0.001 was employed. The HMAP profile-profile alignment did not generate any matches with E-value better than 0.46. Using the most closely matched templates, we constructed LRRK1 models as described above for LRRK2. Again, the top four models as ranked by the statistical potential DFIRE were examined. Three of the four templates were beta-propeller proteins. Nevertheless, even with the beta-propeller repeat templates, there was very little consistency in the models. One of the highest-ranking models for LRRK1 was based on a template for the BUB3 mitotic checkpoint protein (PDB code 1yfq) that was also used for LRRK2. This, combined with the fact that the highest-ranking models were all beta-propeller proteins, suggests a remote relationship between the putative WD40 domain of LRRK1 to the WD40 domain of LRRK2. However, the lack of conservation of the WD repeat motif, absence of any detectible primary sequence relationship between the carboxyl-terminus of each protein, and the variability in the LRRK1 models suggests that LRRK1 and LRRK2 are highly diverged. Moreover, the LRRK2 WD40 has a positively-charged (i.e. basic) cleft (Figure 1A) typical of functional WD40 domains, and a similar positively-charged region was not evident in the LRRK1 models. This analysis was therefore not consistent with the presence of a functional WD40 domain in LRRK1, raising the possibility that this domain plays a role in the neurotoxicity associated with LRRK2 PD mutations.

### The WD40 Domain Is Required for LRRK2 Neurotoxicity

**Figure 2:**
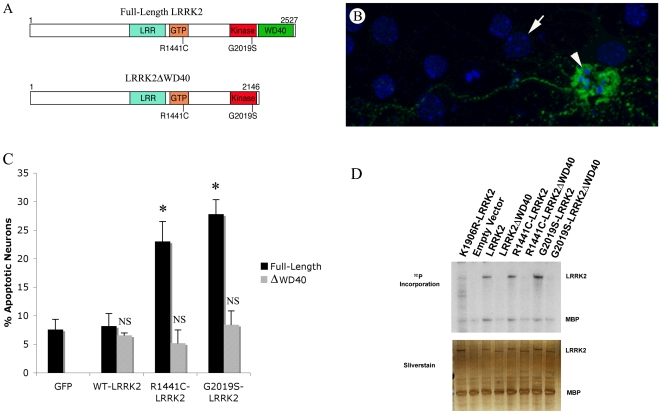
The WD40 domain is critical for LRRK2 neurotoxicity and autophosphorylation. (A) Schematic of LRRK2 and ΔWD40-LRRK2 showing major domains. The WD40 domain was removed by terminating LRRK2 at amino acid 2146. (B) A LRRK2-transfected apoptotic neuron. The arrow indicates a non-apoptotic nucleus, and the arrowhead indicates an apoptotic cell co-transfected with GFP and RC-LRRK2. (C) Removal of the WD40 domain abolishes the neurotoxicity of PD-mutant forms of LRRK2. Wild-type LRRK2 (WT), R1441C LRRK2 (RC), and G2019S LRRK2 (GS) with and without the WD40 domain (ΔWD40) were assessed for neurotoxicity. Murine cortical neurons were co-transfected with LRRK2 constructs and GFP, and the percentage of apoptotic nuclei were assessed 48 hours post-transfection. Data represent the mean±seven individual experiments. Data was assessed using ANOVA followed by Duncan's Multiple Range analysis p<0.05. (D) Removal of the WD40 domain has a differential effect on LRRK2 autophosphorylation and trans-phosphorylation. The different forms of LRRK2 were immunoprecipitated from 293T cells and assessed for their ability to autophosphorylate and to trans-phosphorylate MBP. Top panel is an autoradiogram and bottom panel is silver-stained gel demonstrating the presence of the different forms of LRRK2 and MBP in similar amounts between different conditions.

We explored the role of the WD40 domain in LRRK2 neurotoxicity by testing whether the WD40 is necessary for the LRRK2 PD mutations to elicit neurotoxicity in a well-established LRRK2 neurotoxicity assay [10], [11], [30]. To accomplish this, we first generated wild-type and PD mutant LRRK2 constructs with and without the WD40 domain (WD40 lacking constructs are referred to as “ΔWD”; Fig. 2A), and confirmed that all molecules exhibited similar stability and expressed at similar levels (Supplemental Figure S1). We then transfected mouse primary neuronal cultures with these LRRK2 constructs, or a construct expressing GFP alone, and quantified the percentage of transfected cells (i.e., GFP positive) undergoing apoptosis. Specifically, we transfected neurons with either full length or ΔWD constructs of wild-type, R1441C- or G2019S-LRRK2. Deletion of the WD40 domain completely blocked the neurotoxicity associated with both LRRK2 PD mutations tested (Figure 2C).

### Deletion of the WD40 Domain Eliminates LRRK2 Autophosphorylation

The kinase function of LRRK2 is linked to its neurotoxicity [10], [11], so we next examined whether deleting the WD40 domain alters LRRK2 kinase activity. We performed *in vitro* kinase assays on LRRK2 immunoprecipitated from transfected 293T cells, assessing both autophosphorylation and trans-phosphorylation of the model substrate myelin basic protein (MBP). Strikingly, deletion of the WD40 domain essentially abolished LRRK2 autophosphorylation (Figure 2D). In contrast, the effects on trans-phosphorylation of MBP were more modest, particularly for G2019S LRRK2, for which removal of the WD40 domain had little effect. However, the level of MBP phosphorylation for the WD40-deleted constructs was only slightly greater than for kinase dead LRRK2.

### LRRK2 Forms a Dimeric Complex That Is Lost Upon WD40 Deletion

The striking correlation between loss of autophosphorylation and reduced neurotoxicity led us to further explore the effects of the loss of the WD40 domain on LRRK2 biology. Given the well-known role of WD40 domains in protein-protein interactions, one effect of the ΔWD truncation could be the failure of LRRK2 to interact normally with binding partners. LRRK2 has been reported to be part of a ∼600 kDa complex, as well as of much larger complexes [20]. The size of the larger complex is difficult to estimate accurately, but it is not part of the void volume. Moreover, studies using recombinant fragments of LRRK2 indicate that the ROC-COR region of the protein exists as a dimer that may be disrupted by the R1441C PD mutation [20]. Thus, we next explored whether loss of the WD40 domain alters LRRK2 complex formation.

**Figure 3:**
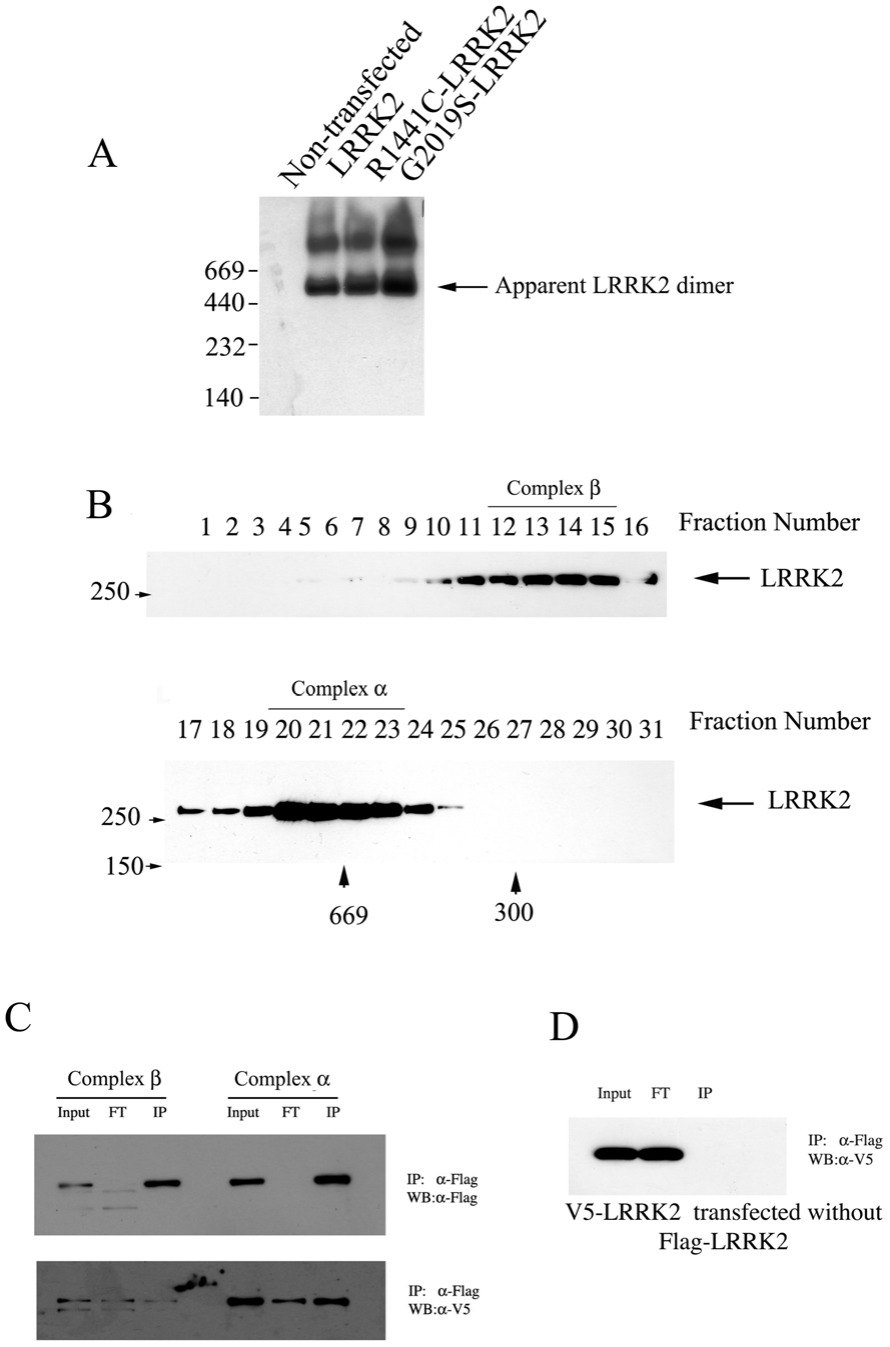
LRRK2 is found in a dimeric and a high molecular weight complex. (A) Native gel electrophoresis of wild type and PD mutant forms of LRRK2. GFP-tagged wild-type, R1441C- and G2019S-LRRK2 were separately transfected into 293T cells and whole cell lysates prepared from these cells were separated by a non-denaturing blue native gel and immunoblotted using anti-GFP antibody. (B) Size-exclusion gel filtration chromatography of wild type and PD mutant forms of LRRK2. Lysates prepared from LRRK2-transfected cells as in (A) were separated by gel filtration chromatography show a similar pattern of two complexes as seen using blue native electrophoresis. (C) The α-complex is a LRRK2 dimer. 293T cells were co-transfected with FLAG- and V5-tagged LRRK2 and separated by size exclusion gel filtration as in (B). Fractions 20–23 (α-complex) and 12–15 (β-complex) were pooled and immunoprecipitated with the anti-FLAG antibody and immunoblotted with anti-FLAG (upper panel) or anti-V5 (lower panel). The amount of V5-LRRK2 that co-immunoprecipitates with the anti-FLAG antibody indicates that the majority, if not all, of the α-complex is a LRRK2 dimer, while the majority of LRRK2 in the β-complex is monomeric. (D) V5-LRRK2 singly transfected and processed as in (C) demonstrates that there is no cross reactivity between anti-FLAG and V5-LRRK2.

To assess LRRK2 complex formation we analyzed lysates from 293T cells transfected with full-length wild type and PD mutant LRRK2 using blue native gel electrophoresis and size exclusion gel filtration. Experiments employing both of these methods demonstrate that LRRK2 is predominantly found in two complexes: a ∼600 kDa “α-complex” and a much larger “β-complex” (Figs. 3A and B). Interestingly, we did not observe monomeric LRRK2, and neither of the LRRK2 PD mutations appeared to alter the formation of these complexes (Fig. 3A; data not shown).

**Figure 4:**
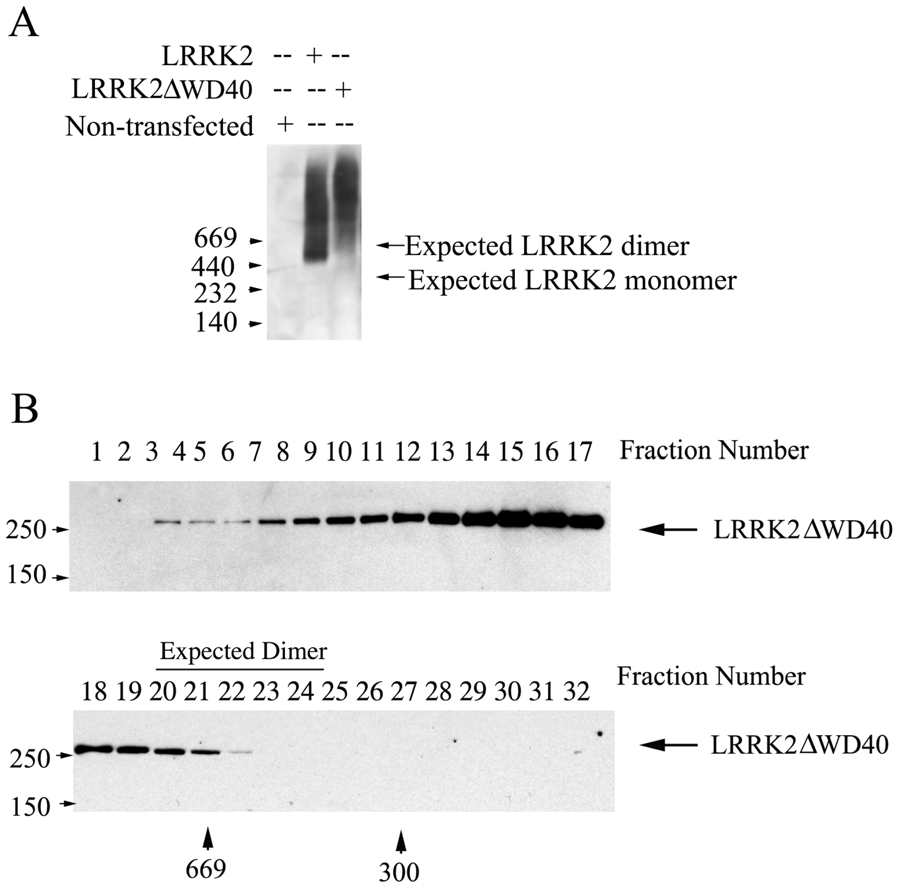
The WD40 domain is necessary for the formation of the dimeric LRRK2 α-complex. (A) Native gel electrophoresis demonstrates absence of the LRRK2 dimer for ΔWD40-LRRK2, and an increase in the high molecular weight LRRK2 immunoreactivity (B) Size-exclusion gel filtration chromatography of ΔWD40-LRRK2 similarly demonstrates a decrease in the α-complex and increase in β-complex.

The absence of monomeric LRRK2 led us to question whether the ∼600 kDa complex was a LRRK2 dimer or monomeric LRRK2 complexed with other proteins with which it has been demonstrated to interact [31], [32], [33]. To address this question we co-transfected 293T cells with two forms of full length LRRK, one tagged with V5 and the other tagged with FLAG, and used immunoprecipitation to analyze pooled fractions containing the α- or β-complexes. IP performed with anti-FLAG antibody demonstrated that the two forms of LRRK2 clearly co-purify from the α-complex, but much less so from the β-complex fractions (Figure 4C). Theoretically, if all LRRK2 in these complexes is dimeric and generated randomly from the two tagged molecules, half of the molecules will be homo-dimers (FLAG-FLAG or V5-V5) while the other half will be heterodimers (FLAG-V5 or V5-FLAG). In this scenario, immunoprecipitation would pull down twice as many molecules corresponding to the IP antibody compared to the other tagged form (i.e., a FLAG pull-down will isolate FLAG-FLAG, FLAG-V5 and V5-FLAG molecules; 4 FLAG molecules and 2 V5 molecules). To determine the relative sensitivity of the V5 and FLAG antibodies, we sequentially probed the same membrane from the FLAG IP, first with V5 (lower gel, Fig. 3C) and then (following stripping) with FLAG (upper gel, Fig. 3C). Densitometry measurements on the input lanes of the α-complex showed that the V5 antibody is ∼1.5X more sensitive than the FLAG antibody (Figure 3C: FLAG input OD = 3.05; V5 input OD = 4.54). We then used this correction factor to correct for the enhanced sensitivity of the V5 antibody by dividing the OD measurement of the V5-probed IP by 1.5. We found that approximately 1.9X more FLAG- than V5-LRRK2 co-immunoprecipitated from the α-complex, indicating that much, if not all, of this complex is dimeric LRRK2 (FLAG-LRRK2 IP OD = 4.95; V5-LRRK2 IP OD: 3.97/1.5 = 2.65; Ratio of FLAG/V5: 4.95/2.65 = 1.87). In contrast, very little LRRK2 was co-immunoprecipitated from the β-complex.

To explore whether there might be a relationship between the presence of LRRK2 in these protein complexes and its neurotoxicity, we tested whether ΔWD40-LRRK2, which lacks neurotoxicity, normally distributes in these complexes. Strikingly, ΔWD40-LRRK2 largely redistributed from the α- to the β-complex when assessed by either blue native gel electrophoresis or size-exclusion gel-filtration chromatography (Figures 4A & B). We did not observe monomeric ΔWD40-LRRK2, and immunoprecipitation of differentially tagged ΔWD40-LRRK2 molecules confirms that it remains able to oligomerize (data not shown).

## Discussion

Previous work demonstrated that LRRK2 can self-associate and is found primarily in a 500–600 kDa complex that was proposed to represent dimeric LRRK2 [18]. However, this work did not exclude the possibility that the 500–600 kDa complex contained monomeric LRRK2 associated with Hsp90, tubulin, or other LRRK2-interacting proteins [31], [32], [33]. In contrast, by performing co-immunoprecipitation experiments selectively on fractions containing the α or β complexes, we demonstrate conclusively that the α complex is composed of a LRRK2 dimer, whereas most LRRK2 in the β complex is monomeric. We did not observe monomeric LRRK2 in blue native gel or gel-filtration chromatography, indicating that LRRK2 may exist as constitutive dimer. Our observations correlating the loss of the LRRK2 dimer and suppression of neurotoxicity raise the possibility that strategies that prevent LRRK2 dimerization may interfere with its function and ability to cause neuron death.

Experiments utilizing fragments of mammalian or bacterial LRRK2 suggest that the pathogenic R1441C LRRK2 mutation disrupts LRRK2 dimerization [18], [24]. In our studies of full length human LRRK2, we did not observe isolated monomeric LRRK2 when studying the wild type, ΔWD40 or R1441C forms of the protein. Moreover, we find that R1441C-LRRK2 distributes between the α and β complexes similarly to the wild type protein. While our experiments employed LRRK2 overexpression, and may therefore not accurately reflect the behavior of endogenous levels of protein, they suggest that if, in the context of the intact protein, the R1441C mutation disrupts dimerization of the ROC domain, other LRRK2-LRRK2 interaction points maintain the dimeric LRRK2 complex. The fact that R1441C-LRRK2 remains dimeric is further consistent with a potential role for this form of LRRK2 in its neurotoxicity.

Previous work on LRRK2 has focused almost exclusively on how functional changes in its GTPase and kinase domains may affect neurotoxicity. However, LRRK2 is a large multi-domain protein and the potential role of other domains in LRRK2 function and ability to cause neurodegeneration is much less well explored. Similar to our findings, previous work demonstrates that deletion of the WD40 domain almost entirely prevents autophosphorylation [16]. In addition, the G2385R polymorphism in the WD40 domain is over-represented in ethnic Chinese patients with PD [34], and this polymorphism increases the sensitivity of cells to hydrogen peroxide [27]. Our structural studies indicate that the carboxyl-terminal regions of LRRK1 and LRRK2 differ considerably (Fig. 1), and these differences may contribute to an explanation of previous work demonstrating the failure of LRRK2 PD mutations to cause neurotoxicity when placed in the context of the LRRK1 protein [22]. While one study suggests that LRRK1 contains a WD40 domain [35], this putative WD40 domain, if it exists, is divergent from a canonical WD40 domain. Homology modeling of the LRRK2 carboyxl-terminus strongly supports the notion that this region forms a WD40 domain, including the presence of a well-defined patch of positively charged residues. In contrast, the LRRK1 carboxyl-terminus is not well modeled as a WD40 domain, and data from human subjects further indicates a lack of selective pressure to maintain key residues [36]. Our observation that PD mutant forms of ΔWD40-LRRK2 do not induce apoptosis indicates that these PD-linked mutations require the WD40 domain to cause neuron death. Consistent with this notion, a previous study demonstrated that deleting the WD40 domain blocks the ability of LRRK2 to activate caspases in SH-SY5Y cells [37]. Our data are consistent with this report, and we advance this finding by assessing toxicity in neurons with an additional PD mutation, and by characterizing the biochemical properties of ΔWD40-LRRK2.

The WD40 domain is a scaffolding structure that is found in a wide range of proteins but is often utilized to produce vesicles and sort cargos [38], [39]. The physiological role of LRRK2 is not well understood, making detailed analysis of the effects of the WD40 on LRRK2 function difficult. The WD40 deletion eliminated the 600 kDa dimer complex and LRRK2 autophosphorylation activity indicating the necessity of the WD40 for the structure and auto-regulatory function of LRRK2. Our findings highlight the importance of the WD40 domain for LRRK2 function in neurons, and indicate that future studies of the WD40 domain may help to elucidate important features of LRRK2 biology relevant to the pathogenesis of Parkinson's disease.

## Materials and Methods

### Structural Modeling

Residues 2101–2527 of LRRK2 were taken to represent the WD40 domain of LRRK2. No suitable modeling templates were found using one iteration of PSI-BLAST [40] and an E-value cutoff of 0.001 so the profile-profile alignment program HMAP [41] was then used to identify possible templates from the Protein Data Bank [42]. Eleven template structures were selected with E-values ranging from 10^−20^ to 10^−24^ for subsequent modeling. Sequence to structure alignment of the WD40 domain to each of these templates was also carried out with HMAP. Models based on each template were constructed using the program NEST [29]. Since there were a number of templates with similar E-values and since the homology was remote, an evaluation of the models themselves was carried out using the statistical potential DFIRE [43] and the top four were structurally aligned to each other to determine whether the models based on different templates were consistent with each other. These models were based on the structures of TUP1 (PDB code 1erj), actin interacting protein 1 (1nr0), cytosolic Fe-S assembly protein 1, (2hes) and BUB3 mitotic checkpoint protein (1yfq). Although there were some differences between the models at the amino and carboxyl-termini, the region for residues 2364–2480 were consistent with each other using the different proteins as models for the LRRK2 WD40.

Residues 1561–2038 were taken to represent the putative WD40 domain in LRRK1. A search of the non-redundant sequence database (nr) using 5 iterations of PSI-BLAST with an E-value cutoff of 0.001 did not identify any similarity to LRRK2 or any of its close homologs. Using the HMAP profile-profile alignment program and a larger E-value cutoff of 10.0 a number of templates were identified, none with E-value better than 0.46, however. Models based on these templates were constructed as described above for LRRK2 and again the top four models as ranked by the statistical potential DFIRE were examined. Although the top three were all beta-propeller proteins, there was very little consistency between the models themselves and the characteristic WD motif was poorly conserved. One of the highest ranking models for LRRK1 was based on a template that was also found for LRRK2, the BUB3 mitotic checkpoint protein (PDB code 1yfq). This, combined with the fact that highest ranking models were all beta-propeller proteins suggests a relationship, however remote, between the putative WD40 domain of LRRK1 to the WD40 domain of LRRK2. But the lack of conservation of the WD motif, the absence of any detectible primary sequence relationship between the WD40 domains of each protein, and the variability in the models of LRRK1 suggests that they are highly diverged. Moreover, the LRRK2 WD40 has a positively-charged (i.e. basic) cleft (Figure 1A), but no similar positively-charged region was evident in the models of LRRK1.

### LRRK2 Neuron Toxicity Assay

Cortical neurons were prepared from 16.5 gestation day mouse embryos as previously described [30], [44]. Using Lipofecatamine 2000 on DIV 4, neurons were transfected with GFP alone or co-transfected with GFP and LRRK2 (WT and mutants as indicated) in a 1∶9 ratio. This ratio is required to ensure expression of LRRK2 in GFP expressing cells, as LRRK2 is a large protein that often expresses poorly. Both N-terminally GFP tagged and un-tagged LRRK2 constructs were used and gave similar results (data set described used amino-terminal tagged GFP LRRK2; untagged data is not shown). The use of GFP-tagged LRRK2 was undertaken because this improves the identification of transfected cells, thus streamlining the counting procedure. The LRRK2 constructs were made using the Gateway system as described previously [30]. Cells were fixed with 4% formaldehyde at DIV 6, blocked for 1 h in 10% goat serum, 0.1% triton-X PBS and probed overnight at 4°C with 1∶1000 anti-GFP polyclonal antibody (#ab6556, Abcam) in blocking solution. The cells were then probed with an anti-rabbit FITC antibody for 1 hour (#711-095-152, Jackson Immuno). Coverslips were mounted with VectaShield/DAPI (#H-1200, Vector Labs). The percentage of GFP-positive neurons with either apoptotic bodies or pyknotic nuclear features was determined as described previously [30]. Data set shown is the average of seven independent experiments.

### Blue Native Electrophoresis

HEK293T cells (8×10^6^ cell/10 cm plate) were transfected using Lipfectamine 2000 according to company protocol (11668027, Invitrogen). The cells were collected in a HEPES lysis buffer: 20 mM HEPES pH 7.0, 150 mM NaCl, 0.1% NP-40, 2 mM EGTA, 10% glycerol, 1 mM DTT, 200 mM Na_2_VO_4_, 10 mM NaF, 25 mM β-glycerophosphate, Complete Protease Inhibitor Cocktail (#11836153, Roche). The cells were disrupted by slowly forcing sample through 21 and 26.5 gauge syringes 5x and 10x respecitvely and centrifuged for 20 minutes at 20,000×g at 4 degrees Celsius. Blue native gel electrophoresis was performed according to the protocols accompanying the blue native gel buffers (#BN2008, Invitrogen). The gel was subsequently incubated in 0.1% SDS for 20 minutes before transferring to a polyvinylidene fluoride (PVDF) membrane. Western blot was performed using previously described procedures [45]. Blots were then probed for GFP-LRRK2 using 1∶2000 anti-GFP mAb (sc-9996, Santa Cruz) in 0.1% Tween 20, 5% milk PBS. Anti-mouse HRP-conjugated secondary antibody was used at 1∶10,000 (#34080, Thermo Scientific) followed by detection with West Pico Supersignal chemiluminescence (#31430, Thermo Scientific).

### Size-Exclusion Gel-Filtration

HEK293T cells (22×10^6^) were plated in a 15 cm dish and transfected the following day using Lipofectame/Plus reagent (#18324/#11514, Invitrogen) according to manufacturer's protocol. The cells were lysed using the HEPES lysis buffer described above in the methods for Blue Native gel electrophoresis, and subsequently homogenized with 20 strokes with dounce and centrifuged 20,000×g for 20 minutes at 4 degrees Celsius. Size-exclusion gel filtration was performed using a GE Superose 10/300 GL Tricorn 6 Column with an AKTA FPLC apparatus. Samples were collected at a flow rate of 0.24 ml/minute in 0.35 ml fractions and subsequently separated on a 7.5% bis-tris polyacrylamide gel. Following transfer to a PVDF membrane, the samples were probed with anti-GFP or anti-V5 antibody (#R960, Invitrogen) as described in the methods for Blue Native gel electrophoresis. The GFP- and V5-tagged LRRK2 constructs were made described previously with the tags placed on the amino terminus [30].

### In Vitro Kinase Assay

2.6×10^6^ HEK293T cells were plated in a 6 cm dish for each condition. Cells were transfected and harvested as described above for Gel Filtration. Immunoprecipitation was performed on a rotator at 4°C. The lysates were then pre-cleared for 30 min with 50 ul of pre-washed protein A agarose beads (#11719408, Roche). GFP-tagged LRRK2 was immunoprecipitated from 1 ml of lysate (2 mg/ml protein concentration) using 1 ul of anti-GFP antibody (#ab6556, Abcam). 50 ul protein A agarose beads was then added to the lysates for 2 h. The beads were then washed gently in 50 ul kinase assay buffer (Tris-HCl pH 7.5, 200 mM Na_2_VO_4_, 5 mM β-glycerophosphate, 1 mM DTT, 10 mM MgCl_2_) by continuously rotating the samples. Labeled ATP and MBP (10 mM Cold ATP, 1 µg MBP, and 0.5 µl γP32-ATP; #BLU502A250UC, Perkin Elmer) were then added and the mixture was incubated at 30°C for 30 min while shaking at 14000 rpm on a thermoshaker (#PMHT, Boekel Grant-Bio). Samples were then separated using SDS-PAGE and silver stained according to Silverquest protocol (#LC6070, Invitrogen).

## Supporting Information

Figure S1 Immunoblot of LRRK2 demonstrating equivalent expression. All (GFP-tagged) constructs with and without PD mutations and the WD40 domain were transfected into 293T to assess expression levels. Immunoblot was stained using anti-GFP and b-tubulin was used as a loading control.
(1.12 MB TIF)
