# Supplementary figures and images for: The WD40 Domain Is Required for LRRK2 Neurotoxicity
Source: PLoS One. 2009 Dec 24;4(12):e8463. doi: 10.1371/journal.pone.0008463 (PMC2794542; doi:10.1371/journal.pone.0008463)

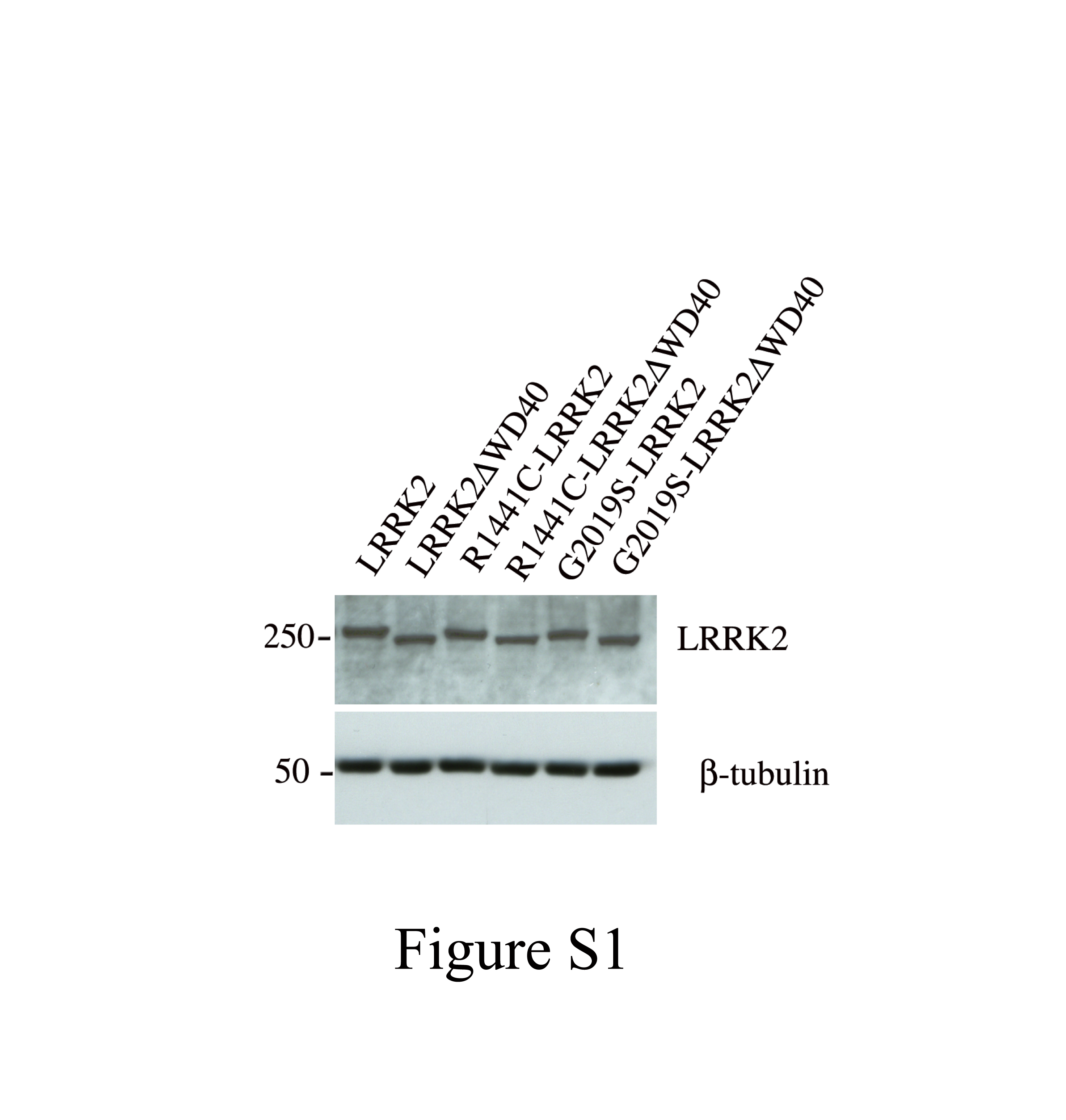

Supplement: Figure S1 — Immunoblot of LRRK2 demonstrating equivalent expression. All (GFP-tagged) constructs with and without PD mutations and the WD40 domain were transfected into 293T to assess expression levels. Immunoblot was stained using anti-GFP and b-tubulin was used as a loading control. (1.12 MB TIF) [file pone.0008463.s001.tif]
